# The Human Papillomavirus (HPV) Laboratory e-Manual: A comprehensive guide for HPV testing and research

**DOI:** 10.1016/j.jcv.2025.105834

**Published:** 2025-07-10

**Authors:** Laila Sara Arroyo Mühr, Carina Eklund, Camilla Lagheden, Rita Mariel Correa, María Dolores Fellner, Maria Alejandra Picconi, Nazlı Songur, Murat Gultekin, Kate Cuschieri, Jean-Luc Prétet, Quentin Lepiller, Alice Baraquin, Steffi Silling, Kristiane Søreng, Milan Stosic, Gro Kummeneje Presthus, Marc Arbyn, Michael Peeters, Steven Van Gucht, Elizaveta Padalko, Taeyang Chin, Kaat Kehoe, Ana Rita Pereira, Nina Redzic, Davy Vanden Broeck, Monica Molano, Gerald L. Murray, Suzanne M. Garland, Ligia A. Pinto, Dianna E. Wilkinson, Mario Poljak, Joakim Dillner

**Affiliations:** a International HPV Reference Center, Center for Cervical Cancer Elimination, F56, Karolinska Institutet and Karolinska University Hospital, 141 86 Stockholm, Sweden; b Oncogenic Viruses Service, Argentine HPV Reference Laboratory, National Institute of Infectious Diseases-ANLIS “Dr. Malbrán”, Vélez Sarsfield 563 (C1282AFF), Buenos Aires, Argentina; c Hacettepe University Faculty of Medicine, Department of Obstetrics and Gynecology, Division of Gynaecological Oncology, Ankara, Turkey; d Scottish HPV Reference Laboratory, Department of Laboratory Medicine, Royal Infirmary of Edinburgh, EH16 4SA & HPV Research Group, Centre for Reproductive Health, University of Edinburgh, UK; e CHU Besançon, French National Papillomavirus Reference Center, F-25000 Besançon, France; f Université Marie et Louis Pasteur, CHU Besançon, CNRS, Chrono-environnement (UMR 6249), F-25000 Besançon, France; g German National Reference Center, Institute of Virology, University Hospital and University of Cologne, Fuerst-Pueckler-Str. 56, 50935 Cologne, Germany; h Norwegian HPV Reference Laboratory, Department of Microbiology and Infection Control, Akershus University Hospital, Lørenskog, Norway; i The Belgian Reference Centre for Human Papillomavirus, Sciensano, Infectious Diseases in Humans, Viral Diseases, Brussels, Belgium; j The Belgian Reference Centre for Human Papillomavirus, University Hospital Ghent, Laboratory of Medical Microbiology, Ghent, Belgium; k The Belgian Reference Centre for Human Papillomavirus, Algemeen Medisch Laboratorium - Sonic Healthcare Benelux, Antwerp, Belgium; l Centre for Women’s Infectious Diseases, The Royal Women’s Hospital, Melbourne, Victoria, Australia; m Department of Obstetrics, Gynaecology and Newborn Health, University of Melbourne, Parkville, Victoria, Australia; n Murdoch Children’s Research Institute, Melbourne, Victoria, Australia; o Vaccine, Immunity and Cancer Directorate, Frederick National Laboratory for Cancer Research, Frederick, MD 21702, USA; p Viral Vaccines—Science, Research and Innovation, Medicines and Healthcare Products Regulatory Agency, South Mimms, Hertfordshire EN6 3QG, UK; q The Slovenian Reference Center for Human Papillomavirus, Institute of Microbiology and Immunology, Faculty of Medicine, University of Ljubljana, Slovenia

**Keywords:** Quality assurance, Standardization, Cervical screening, Vaccination, Reproducibility

## Abstract

**Background:**

Human Papillomavirus (HPV) vaccination and HPV-based cervical cancer screening are central pillars of the World Health Organization (WHO) global cervical cancer elimination strategy. The WHO HPV Laboratory Manual, published in 2009, has provided essential guidance to promote an internationally comparable quality of HPV testing for many years. As the development in this area is rapid, the Global Network of National HPV Reference Laboratories considered that there is a need for an updated HPV Laboratory e-Manual to serve as a comprehensive and interactive resource for professionals engaged in quality-assured HPV testing for research and/or HPV-based cancer control.

**Content:**

The HPV Laboratory e-Manual covers key areas, including laboratory quality assurance, HPV taxonomy and risk association, collection and handling of specimens, nucleic acid extraction, HPV detection and typing, HPV serology, data management, and the use of international standards. It provides up-to-date protocols and best practices to enhance accuracy and reliability of HPV testing. Interactive features allow for real-time updates, making it a dynamic resource for laboratories worldwide. The e-Manual is freely available at: https://www.hpvcenter.se/hpv-laboratory-manual/.

**Collaborators:**

The e-Manual has been developed by international experts from 11 countries, including contributors from the International HPV Reference Center (IHRC, Sweden), the CDC’s Global HPV Reference Laboratory (USA), and multiple National HPV Reference Laboratories (NRLs). The standard procedure for writing a chapter was that 2 NRLs authored the chapter and 1 other NRL reviewed it.

**Conclusion:**

The HPV Laboratory e-Manual represents a step toward global harmonization in laboratory methodologies for HPV testing, underpinning both research and cervical cancer control efforts.

## 1. Background

The global landscape of HPV research and prevention has advanced significantly since the release of the World Health Organization (WHO) Human Papillomavirus (HPV) Laboratory Manual in 2009 [1]. HPV vaccination and HPV-based screening have become key pillars of the WHO strategy for global cervical cancer elimination [2]. New scientific discoveries and laboratory practices have emerged, underscoring the need for updated resources. To address this, the Global Network of National HPV Reference Laboratories (HPV LabNet) decided to develop an HPV Laboratory e-Manual to offer an interactive and user-friendly guide that reflects current best practices and integrates the latest scientific advancements in HPV research, diagnostics, and cancer control. The aim of this project was to serve as an essential resource for laboratory professionals, researchers, and clinicians worldwide, ensuring high-quality and standardized approaches in HPV testing and prevention efforts globally. The e-Manual is designed as a living document that will continuously evolve, with additional chapters and updates reflecting emerging knowledge and advancements in the field.

## 2. HPV Laboratory e-Manual content

The HPV Laboratory e-Manual provides comprehensive, up-to-date coverage of laboratory procedures essential for HPV research and testing which we hope are relevant for laboratories provided service and research-based HPV testing. It is organized into detailed sections that cover aspects of HPV testing (and associated quality practices) to support clinical, research and epidemiological work. As of 2025-02-19, the manual includes the following chapters:

- Chapter 1: Introduction and Overview: This section provides a general introduction to HPV, its pivotal role in the development of cervical cancer, and outlines the manual’s objectives, structure, and intended use for researchers and clinicians alike.
- Chapter 2: HPV Types: This section offers a thorough exploration of the diverse HPV genotypes, detailing their specific roles in cancer progression in relation to cervical, and other ano-genital and oropharyngeal cancers. It includes a breakdown of how HPVs are classified within its broader viral family and special attention is given to the distinction between established and non-established HPV types. This section is crucial for understanding the molecular heterogeneity of HPV, the pathogenesis of HPV-related cancers, and the continued efforts to identify and categorize emerging HPV strains.
- Chapter 3: Laboratory Quality Assurance: This section comprises best practices for ensuring rigorous quality control in laboratory testing, with a particular focus on maintaining consistency, accuracy, and reliability of HPV diagnostics in both research and clinical environments. The chapter highlights the importance of a well-structured Quality Management System (QMS), including adherence to standards like ISO 15189, and emphasizes continuous improvement across preanalytical, analytical, and post-analytical stages of testing.
- Chapter 4: Collection and Handling of Specimens: This section offers a guide on procedures for collecting, storing, and transporting specimens for HPV testing, ensuring sample integrity, and minimizing contamination risks. This chapter covers handling techniques for various sample types (including cervical exfoliated cells, self-collected swabs, urine specimens, male external genital specimens, anal swabs, oral rinses, formalin-fixed paraffin-embedded specimens, and serum) to support accurate testing and offers suggestions for minimizing potential errors.
- Chapter 5: Nucleic Acid Extraction: This section provides detailed protocols for efficiently extracting nucleic acids from various biological samples. It focuses on techniques that ensure accurate, reproducible results in diagnostic testing. The chapter addresses challenging sample types, such as FFPE material, as well as novel ones like urine specimens, and describes innovative approaches including cell-free DNA extraction.
- Chapter 6: HPV detection and typing: This section covers a comprehensive range of techniques for HPV detection and typing, including detailed protocols for HPV amplification, as well as for emerging technologies (Next Generation Sequencing and digital PCR). It also provides guidance on assay selection by discussing testing goals, equipment needs, assay complexity, data management, cost considerations, the differences between in-house and commercial assays, and regulatory issues related to laboratory-developed tests. Two appendices are included: Appendix I contains primer sequences, and Appendix II offers an example protocol for fully validated in-house developed assay (RIATOL quantitative real-time PCR (qPCR)).
- Chapter 7: Serology: This section provides in-depth information on serological methods, including ELISA and neutralization assays, for detecting HPV antibodies in patient samples. It aids researchers and clinicians in understanding immune responses and assessing the risk of disease progression in HPV-infected individuals. Additionally, the manual includes two appendices: Appendix 1 details the VLP synthesis procedure, while Appendix 2 presents comprehensive standard operating procedures (SOPs) for a multiplex antibody binding assay using Luminex xMAP technology.
- Chapter 8: International Standards and Secondary Standards: This section includes a discussion on the use of internationally recognized HPV standards and secondary reference materials, which are essential for ensuring consistency and comparability across global research studies and clinical diagnostics.
- Chapter 9: Data Management: This section includes practical recommendations for the management of research data, including best practices for data storage, documentation, and sharing, ensuring that all data handling procedures uphold the integrity, security, and confidentiality of data.
- Chapter 10: International and National HPV Reference Laboratories: This section presents a comprehensive list and contact information for the HPV reference laboratories that comprise the Global HPV Reference Laboratory Network, providing resources for collaboration and support in HPV testing and research efforts worldwide.

## 3. Manual features and future updates

Interactive features have been integrated into the e-Manual, allowing for real-time updates as new scientific advancements emerge and research practices evolve. These features make the manual adaptable and ensure it remains at the cutting edge of HPV-related diagnostics. Designed with the user in mind, the e-Manual includes clickable sections and detailed standard operating procedures (SOPs) to enhance the learning experience and improve practical application. In addition, Chapter 2 is structured as an online course featuring videos and practical examples, further enriching the resource (available at: https://www.hpvcenter.se/e-learning-resources/). Moreover, new chapters, such as “Setting up an HPV Laboratory” and “Target Profile Products (TPP)”, are in preparation and will be available in due course. Thus, the manual will be providing an expanding resource for HPV research and cancer control, underscoring its dynamic nature. Users are encouraged to check the manual regularly for the latest updates.

The full manual is freely available at https://www.hpvcenter.se/hpv-laboratory-manual/.

## 4. Collaborators

The HPV Laboratory e-Manual was developed by a group of experts from the Global HPV Reference Laboratory Network, a collaboration of national HPV reference laboratories from around the world. Originally established by WHO in 2005 to support the introduction of HPV vaccines and to monitor disease and infection [3], the LabNet is today a network of national reference laboratories appointed by their respective countries. In recognition of WHO’s Global Strategy for Cervical Cancer Elimination, adopted in 2018, the LabNet’s mission has expanded to also include the development of laboratory expertise and infrastructure for HPV testing in cervical cancer screening. This manual is an outcome of these efforts, providing standardized protocols and best practices for HPV diagnostics and research in support of global cervical cancer elimination initiatives. Each chapter lists the authors responsible for its content.

## 5. Conclusion

The HPV Laboratory e-Manual represents a significant advancement in HPV research and cervical cancer control. By providing up-to-date methodologies, protocols, and interactive features, the e-Manual offers an accessible and dynamic resource for professionals in HPV diagnostics, vaccine research, and cancer prevention. Its open-access model ensures use by researchers, clinicians, and public health professionals and students worldwide, facilitating the standardization and improvement of HPV testing and research. As HPV research continues to evolve, the manual will be updated to reflect the latest scientific discoveries and best practices. By improving the quality and consistency of HPV testing and research, the e-Manual plays an essential role in supporting the global effort to eliminate cervical cancer.

## Data Availability

This article does not involve the use of original research data. The HPV Laboratory e-Manual is publicly available and can be accessed at: https://www.hpvcenter.se/hpv-laboratory-manual/.
